# Rescue endoscopic ultrasound-guided hemostasis for massive gastric cancer bleeding after transcatheter arterial embolization

**DOI:** 10.1055/a-2780-6024

**Published:** 2026-02-17

**Authors:** Hao Zhuang, Jialiang Huang, Duanmin Hu, Guilian Cheng

**Affiliations:** 1 105860 Department of Gastroenterology, Second Affiliated Hospital of Soochow University, Suzhou, China

**Fig. 1:**
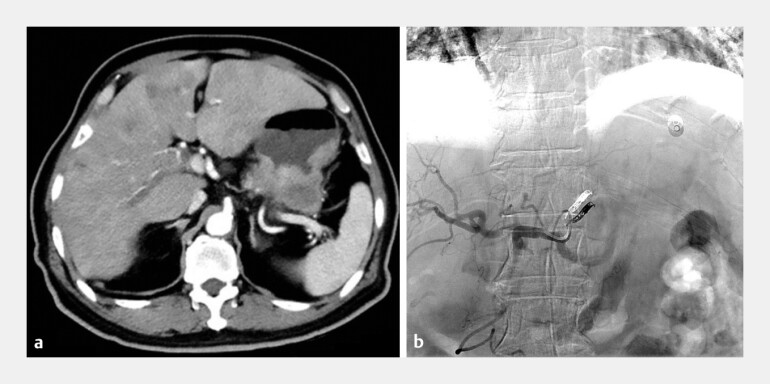
Baseline clinical characteristics of the patient. **a** Enhanced computed tomography (CT) confirmed the tumor extending from the cardia to the gastric body with several slightly enhanced metastasis in the liver. **b** Digital subtraction angiography (DSA) post-TAE observed successfully occlusion in downstream of the left gastric artery. TAE, transcatheter artery embolization.

A 66-year-old man was admitted for massive gastrointestinal bleeding due to advanced gastric cancer with liver metastasis. He had undergone transcatheter artery embolization (TAE) of the left gastric artery for initial tumor hemorrhage 1 month prior ( Fig. 1 ). Given the poor efficacy of conventional endoscopic therapy to diffuse tumor bleeding 1 and the patient refused surgery, emergency endoscopic ultrasound (EUS)-guided hemostasis was performed after careful communication with the patient and his family.

**Fig. 2:**
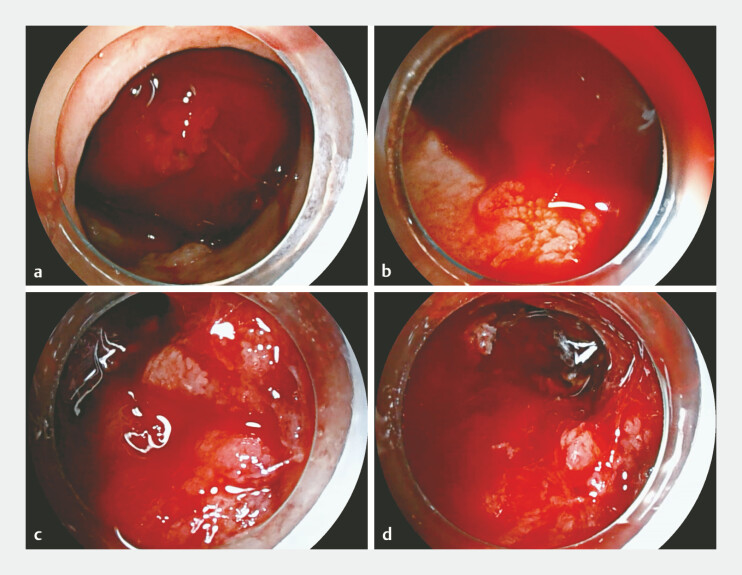
Esophagogastroduodenoscopy (EGD) revealed diffuse active bleeding from the gastric tumor. **a** The huge tumor mixed with the blood clot occupied the whole gastric lumen. **b** – **d** Multiple active bleeding points distributed across the base of cancer.

**Fig. 3:**
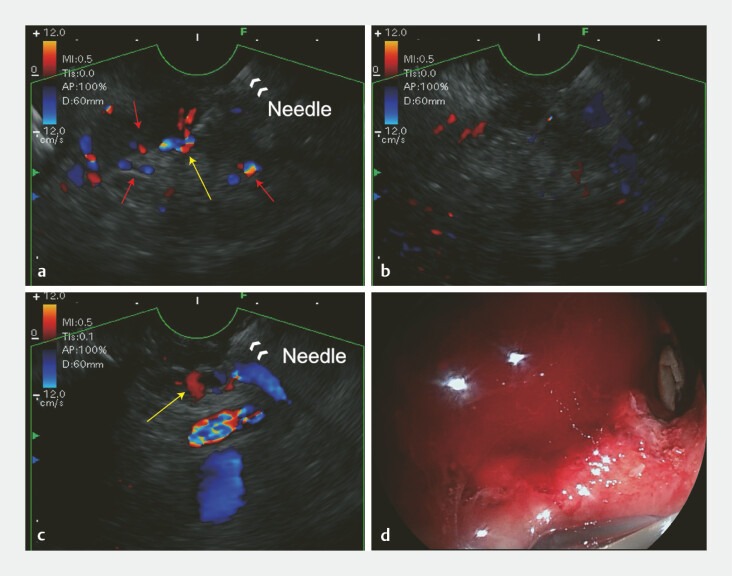
EUS-guided cyanoacrylate injection for massive gastric cancer bleeding. **a** Color Doppler identified a feeding vessel (yellow arrow) providing blood to offending microvascular (red arrow) and EUS-guide cyanoacrylate injection was performed with a 22-gauge needle. **b** Blood flow signals of the feeding vessel and its branches immediately disappeared after injection. **c** Another feeding vessel (yellow arrow) was identified and successfully embolized with the same procedure. **d** No further active bleeding appeared during 10-minute EGD observation. ECD, esophagogastroduodenoscopy; EUS, endoscopic ultrasound.

Under general anesthesia with tracheal intubation, esophagogastroduodenoscopy (EGD) was first performed and revealed a huge tumor extending from the cardia to gastric body, with active bleeding point distributing across the tumor base ( Fig. 2 ). A linear-array echoendoscope with color Doppler was then employed and observed multiple offending microvascular within submucosa. At the convergence point of these microvascular, a large vessel was identified as the dominant feeding vessel, providing major blood flow to the branches. Under EUS guidance, the feeding vessel was punctured with a standard 22-gauge needle. The intravascular location of the needle was confirmed by the injection of 0.5 mL of injection, followed by the administration of 1mL of cyanoacrylate, which resulted in the immediate disappearance of flow signals in the feeding vessel and its branches. Another large feeding vessel was identified at the gastric body and successfully embolized following the same procedure. After operation, repeat >EGD confirmed that no sustained bleeding occurred during 10-minute observation ( Fig. 3 , Video 1 ). The patient was discharged on postoperative day 8 without adverse events or complications. No recurrent bleeding occurred within 1 month follow-up.

EUS-guided cyanoacrylate injection for massive gastric cancer bleeding after TAE. EUS, endoscopic ultrasound; TAE, transcatheter artery embolization.Video 1

EUS-guided hemostasis reported success rates of 78–100% in nonvariceal refractory bleeding 2 3 . Han Chaoqun et al. reported a successful case of EUS-guided lauromacrogol injection for refractory gastric cancer bleeding 4 . To our knowledge, rescue EUS-guided hemostasis for massive gastric cancer recurrent bleeding after TAE have not been reported before.

Endoscopy_UCTN_Code_TTT_1AS_2AB
